# First report of rhino-orbital mucormycosis caused by *Syncephalastrum racemosum* in a diabetic patient with COVID-19 in Iran and review of recent literature

**DOI:** 10.18502/cmm.8.2.10333

**Published:** 2022-06

**Authors:** Mojtaba Taghizadeh Armaki, Jalal Jafarzadeh, Saeid Mahdavi Omran, Masoumeh Bayani, Ali Tavassoli, Leila Faeli, Mohsen Nosratabadi, Sanaz Yaalimadad, Bahador Nikoueian, Iman Haghani, Maryam Moazeni, Tahereh Shokohi, Mohammad Taghi Hedayati, Mahdi Abastabar

**Affiliations:** 1 Department of Medical Mycology and Parasitology, School of Medicine, Babol University of Medical Sciences, Babol, Iran; 2 Infectious Diseases and Tropical Medicine Research Center, Health Research Institute, Babol University of Medical Sciences, Babol, Iran; 3 Clinical Research Development Unit of Babol University of Ayatollah Rouhani Hospital, Babol University of Medical Sciences, Babol, Iran; 4 Department of Medical Mycology, School of Medicine, Mazandaran University of Medical Sciences, Sari, Iran; 5 Invasive Fungi Research Center, Communicable Diseases Institute, Mazandaran University of Medical Sciences, Sari, Iran

**Keywords:** Mucormycosis, *Syncephalastrum racemosum*

## Abstract

**Background and Purpose:**

Invasive mucormycosis is a rare mycosis that affects most cases of uncontrolled diabetes and has a high mortality rate. Patients with COVID-19 are at high risk of developing invasive mucormycosis due to the consumption of anti-inflammatory drugs such as corticosteroids and dexamethasone. *Rhizopus* species followed by *Rhizomucor* spp. and *Mucor* spp. are the main common etiological agents of rhino-orbital mucormycosis. Therefore, this study aimed to present a case of mucormycosis due to *Syncephalastrum racemosum* in a diabetic patient with COVID-19 for the first time in Iran.

**Case report:**

A 73-year-old diabetic female was referred to Ayatollah Rouhani Hospital in Babol, Iran, with a confirmed COVID-19 diagnosis, based on positive RT-PCR and computed tomography of the lungs. She has received methylprednisolone due to severe lung complications. Nasal involvement and left orbital swelling were observed 20 days after the hospitalization. By sinus endoscopic surgery, debridement was done and histopathology indicated wide hyphae (without septa). The sequenced PCR products displayed *Syncephalastrum racemosum*. In the antifungal susceptibility test, amphotericin B showed good activity against *S. racemosum* and the patient survived with timely treatment.

**Conclusion:**

This is the first case report of rhino-orbital mucormycosis due to *S. racemosum* in COVID-19 patient; therefore, *S. racemosum* can be considered one of the etiological factors of rhino-orbital mucormycosis in COVID-19 cases.

## Introduction

Severe acute respiratory syndrome coronavirus 2 (SARS-CoV-2), a highly pathogenic coronavirus that emerged in 2019, has been responsible for a terrible pandemic of an acute respiratory disorder, named ‘coronavirus disease 2019’ (COVID-19) [ 1 ]. Recently, an increase has been reported in the prevalence of some fungal infections such as mucormycosis, aspergillosis, and candidiasis in patients with COVID-19 [ 2 ]. In the case of patients with COVID-19, the use of corticosteroids, such as prednisolone and dexamethasone, is recommended to modulate the inflammatory response, reduce lung damage, and prevent the spread of lung failure [ 3 ]. These therapies can prepare the condition for the presence of human opportunistic fungal pathogens such as Mucorales order. 

Mucormycosis infection in the condition of the COVID-19 pandemic has been reported as a fulminant and lethal infection [ 2 ]. 

*Rhizopus* spp., *Lichtheimia* spp., *Mucor* spp., and *Rhizomucor* spp. are frequently involved in mucormycosis, along with the fungi of the genus *Syncephalastrum* spp., *Saksenaea* spp., *Apophysomyces* spp., and *Cunninghamella* spp. [ 4 ]. Nowadays, the genus Syncephalastrum comprises three species, including *S. racemosum*, *S. monosporum*, and *S. contaminatum* [ 5 ]. *S. racemosum* has been recovered in several cases in patients without COVID-19 [ 4 ]. 

A rhino-orbital mucormycosis infection begins in the nasal cavities and progresses to rhinosinusitis, pharyngitis, and ocular cellulitis with necrosis, ulcers, and black discharges [ 6 ]. Early diagnosis and treatment of mucormycosis are important and affect the outcome of treatment, as it increases the patient’s chances of survival, reduces the need for surgery, and causes less pain for the patient [ 7 ]. Muthu *et al*. (2021) reported 2,568 patients with COVID-19-associated mucormycosis (CAM) in cases with diabetes mellitus (DM) [ 8 ]. Rudramurthy *et al*. (2021) described that uncontrolled DM and high doses of corticosteroid treatment are the principal predisposing factors for this surge [ 9 ]. This study reports *S. racemosum* as a causative agent of rhino-orbital mucormycosis in a COVID-19 patient with uncontrolled DM.

## Case report

**Figure 1:**
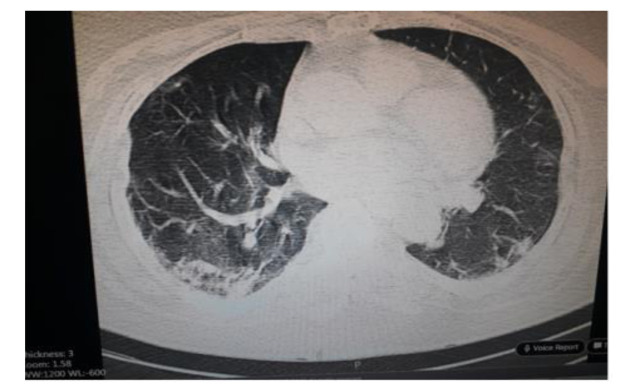
Chest computed tomography scan of a patient with COVID-19

A 73-year-old woman was admitted to Ayatollah Rouhani Hospital in Babol, Iran, on May 15, 2021, with such clinical symptoms as high fever, sore throat, body aches, anorexia, decreased sense of smell and taste, and respiratory problems, as well as the pulse rate of 65 rpm, blood pressure of 110.70 mmHg, and 95% oxygen saturation at admission. The patient had a 10-year history of DM, and her blood sugar level and HbA1c were 305 mg/dL and 10.8%, respectively. One day after admission, a computed tomography (CT) scan of the chest showed evidence of lung involvement (Figure 1) and a real-time polymerase chain reaction (RT-PCR) confirmed the COVID-19 infection (cycle threshold value=27). An infectious disease specialist monitored the necessary clinical functions, from admission to June 20, 2011, to assess the patient’s condition and treatment progress (Table 1). Standard treatment protocols were implemented for COVID-19 treatment management. Initially, the patient stopped taking glibenclamide, and later it was replaced with Zipmet at a dose of 50/1000. Remdesivir injection was started with a daily dose of 200-100 mg along with interferon beta-1a every day in the first week. Dexamethasone 4 mg twice daily for two weeks with heparin, ceftriaxone, and Lasix were prescribed as well. Moreover, prednisolone 25-175 mg daily was also administered during hospitalization.

**Table 1:** The demographic data of the patient during hospitalization

Investigations	At presentation (15 May)	20 May	26 May	4 June	11 June	20 June
WBC	5.2 × 10^3^	6.8 × 10^3^	24.9 × 10^3^	20 × 10^3^	9.3 × 10^3^	8.4 × 10^3^
Neutrophil count (%)	77.7	72	88	80.7	79.8	68.1
Lymphocyte count (%)	19.2	28	9	9.5	13.2	18.4
RBC	5.89 × 10^6^	6.21 × 10^6^	6.69 × 10^6^	5.19 × 10^6^	4.16 × 10^6^	4.36 × 10^6^
Hemoglobin, g/Dl	11.1	11.7	12.5	9.5	8.3	8.3
Platelet count, /lL	152 × 10^3^	228 × 10^3^	329 × 10^3^	248 × 10^3^	163 × 10^3^	232 × 10^3^
Alkaline phosphatase, IU/L	153	185	189	135	134	172
S.G.O.T (AST)	24	32	17	26	30	22
S.G.P.T	19	22	13	21	27	15
BUN	ND	25	28	32	24	20
Serum creatinine, mg/dL	ND	0.9	11	1.3	2.3	1.3
CRP	47	90	29	ND	ND	ND
PTT (Sec)	60	30	35	115	43	40
IL-6 (pg/ml)	50	19	ND	ND	ND	ND
D-dimer (ng/ml)	401	ND	ND	ND	ND	ND
PO2 (%)	95	93	96	95	97	95
Blood pressure	110/70	130/90	120/8	130/80	120/80	150/90
Heart rate	65	53	58	70	72	70
Respiratory rate	18	18	19	20	18	18

ND; not determined

On the sixth day of hospitalization, blood sugar reached 338 mg/dL and 8 units of insulin were administered daily for one week. On the ninth day of hospitalization, carotid Doppler ultrasonography of the artery of the neck and eyes showed no evidence of thrombosis and dissection. However, evidence of mild atherosclerosis was observed in the form of calcified plaques in the left carotid bulb. Treatment with Amphotericin B was started at a single dose of 150 mg/kg/day and continued until the last day of hospitalization at a dose of 250 mg/kg/day. 

**Figure 2:**
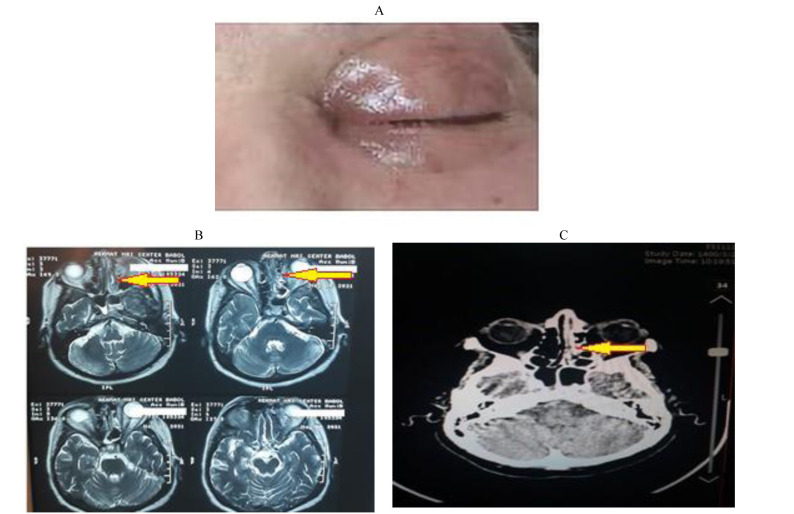
A; Preoperative presentation of left eye proptosis and chemosis; B; MRI scan image showing the involvement of left eye; C; MRI scan image showing the involvement of paranasal sinuses

On the 20th day of hospitalization, the patient complained of swelling and pain in the left eye and nasal involvement (Figure 2). The clinical examination of the eye revealed proptosis, chemosis, and cellulitis around the eye, as well as internal vision limitation. The complications in oculus sinister (OS) were observed in the left eye. Magnetic resonance imaging (MRI) of the eye and paranasal sinus showed drooping of the upper eyelid of the left eye, indicating the involvement of the upper branch of the III nerve, as well as hypertropia or exotropia in the patient’s eye (Figure 2). The forehead and mouth fold shifted to the right due to the seventh cranial nerve (CN VII) involvement. The most important ocular problem was the presence of a cherry-red spot indicating the involvement of the central retinal artery [ 10 ]. Moreover, turbidity and thickening of the mucosa of the right frontal sinuses, maxilla, sphenoid, ethmoid, and effusion of the left sphenoid sinus were detected in the paranasal sinuses (Figure 2).

**Figure 3:**
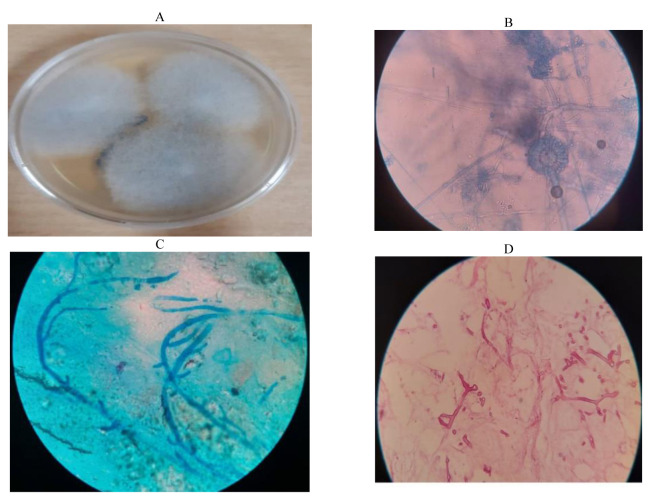
A; Culture of biopsy sample and growth of *S. racemosum* colony on Sabouraud’s dextrose agar at 35 ºC, B; Direct microscopic examination of *S. racemosum* with staining lactophenol cotton blue, C; Biopsy section stained with methylene blue stain showing typical aseptate hyphae, and D; Histopathological section stained with H&E stain showing broad aseptate fungal hyphae

On the 30th day of hospitalization, the patient underwent endoscopic sinus surgery and removal of blackish necrotic tissue. The biopsy specimens were referred to the department of histopathology and mycology. During the second surgery, on the 37th day of hospitalization, the patient’s sinuses were drained during surgery to facilitate treatment. In the first and second samples, a part of the biopsy specimen was inoculated on Sabouraud dextrose agar supplemented with chloramphenicol (SC, Merck, Germany) (Figure 3) and incubated at 37 °C. After two days of incubation, a small colony of fungal growth was observed in the culture (Figure 3-A). The colony was examined under a microscope after staining with lactophenol cotton blue (Figure 3, B). On biopsy and histopathology examination, aseptate, broad, and ribbon-shaped hyphae were observed in the tissue sections stained with methylene blue and H&E (Figures 3, C, and D).

The DNA extraction and molecular identification at the species level were performed by sequencing the ITS rDNA (ITS1 5'-TCCGTTGGTGAACCAGCGG-`3 and ITS4 5'-TCCTCCGCTTATTGATATGC-`3), as previously described [ 11 ]. The DNA sequence of the ITS rDNA region (accession number OL636400) showed 100% similarity with *S. racemosum* (KX815982).

Intravenous liposomal Amphotericin B (1 mg/kg/ day) was initiated on day five and the patient underwent surgery on day 37 with excision of necrotic tissue.

The patient’s condition was stable during the follow-up seven days after the complete evacuation of the sinuses. Amphotericin B was stopped and posaconazole oral suspension (80 mg/day) resumed for 30 days.

*In vitro* antifungal susceptibility testing of the *S. racemosum* was done according to the recommendations stated in the Clinical and Laboratory Standards Institute (CLSI) M38-A3 guidelines [ 12 ]. The minimum inhibitory concentrations (MICs) were 0.032 μg/ml for amphotericin B, 0.25 μg/ml for luliconazole, 0.5 μg/ml for posaconazole, 0.5 μg/ml for itraconazole, 4 μg/ml for tavaborole, 1 μg/ml for econazole, 1 μg/ml for isavuconazole, 2 μg/ml for caspofungin, 4 μg/ml for ketoconazole, 4 μg/ml for natamycin, ≥4 μg/ml for terbinafine, ≥4 μg/ml for butenafine, ≥16 μg/ml for voriconazole, ≥16 μg/ml for miconazole, ≥16 μg/ml for efinaconazole, ≥64 μg/ml for fluconazole, ≥16 μg/ml for sertaconazole, ≥16 μg/ml for tioconazole, ≥16 μg/ml for ravuconazole. Minimum effective concentration (MEC) values were 1 μg/ml for anidulafungin, and 4 μg/ml for micafungin.

## Discussion

Rhino-orbital zygomycosis is a serious life-threatening fungal infection in cases with diabetes mellitus. The infection can occur following inhalation and deposition of sporangiospores in nasal turbinates [ 13 ]. The development of the disease from paranasal sinuses is either straight or through the vascular occlusion. Infection can spread to the eye through the angular and lacrimal vena [ 14 ]. It spreads intracranially by invading the cavernous sinus and the apex of the orbit (superior orbital fissure ), ophthalmic artery vessels, and cribriform plate ethmoid bone and rarely invades the carotid artery [ 15 ]. 

The clinical findings reported in cases with rhino-orbital zygomycosis include pain, fever, headache, vision loss, chemosis, proptosis, ophthalmoplegia, and facial paralysis [ 4 , 13 ]. The main common agent involving rhino-orbital zygomycosis is *Rhizopus* spp., followed by *Rhizomucor*. Other agents which have been reported to cause rhino-orbital zygomycosis include *Absidia* spp., *Mucor*, *Cunninghamella*, Saksenia, and *Syncephalastrum* [ 4 ]. 

In the present case report, we described a case of rhino-orbital zygomycosis due to *S. racemosum* in an elderly diabetic woman who was previously treated with high doses of corticosteroid for the prevention of lung inflammation following COVID-19 infection. 

Several studies announced that corticosteroids have been associated with lower mortality rates in patients with COVID-19; however, they have increased nosocomial fungal infections [ 4 ]. The results of observational studies were in concordance with those obtained in the present study. The literature review showed many cases of patients with secondary causes of zygomycosis named COVID-19-associated mucormycosis (CAM) [ 16 ] with pulmonary, gastrointestinal, and rhino-orbital disorders [ 17 ]. 

According to Table 2, *S. racemosum* rhino-orbital infection was first reported in a middle-aged man following hepatic cirrhosis in 2008 [ 18 ]. Subsequently, six cases have been documented. The summarized data of cases are presented in Table 2.

**Table 2:** Reported cases of rhino-orbital zygomycosis caused by *S. racemosum*

Authors [ 27 ]	Age/Gender	Country	Underlying condition	Therapy	Outcome	References
Baradkar *et al*. [2008]	45/M	India	Hepatic cirrhosis	Surgical debridement, Amphotericin B	Survived	[ 28 ]
Gomez MZR *et al*. Unpublished case [2011]	64/F	United States	Relapsed ALL after allogenic hematopoietic stem cell transplantation	Liposomal Amphotericin B, granulocyte transfusions and G-CSF	Survived	[ 29 ]
Mathuram *et al*. [2013]	63/M	India	Diabetes mellitus	Amphotericin B	Survived	[ 15 ]
Jayaprakash Rao *et al*. [2016]	58/F	India	Diabetic ketoacidosis	Surgical debridement, Amphotericin B	Survived	[ 30 ]
Singh *et al*. [2021]	40/M	India	Diabetes	Endoscopic sinus Debridement with topical LAmb. Intravenous LAmb 300 mg daily OD for 3 weeks and later shifted to voriconazole 200 BD	Survived	[ 23 ]
	40/M		Diabetes	Anterior and posterior Ethmoidectomy with orbital exenteration and Lamb for 3 weeks	Survived	
Gulati *et al*. [2021]	13/F	India	Aplastic anaemia	Amphotericin B, 25 mg/day	Died	[ 14 ]
Taghizadeh *et al*. [2021]	73/F	Iran	Diabetes/COVID-19	Liposomal Amphotericin B	Survived	

We isolated *S. racemosum* for the first time in a diabetic patient with COVID-19; however, there have been different reports of this species in non-COVID-19 cases in some countries such as India, Italy, Serbia, and the USA [ 14 ].

Gulati *et al*. isolated *S. racemosum* from pansinusitis in a 13-year-old patient with aplastic anaemia [ 14 ] and Pavlovic *et al*. [ 19 ] reported onychomycosis caused by *S. racemosum* in Serbia in a patient subjected to injury during a soccer game.

Basically, treatment of zygomycosis consists of surgical debridement, antifungal therapy, and control of the underlying diseases. Usually, liposomal Amphotericin B and posaconazole are the two most common choices used for zygomycosis [ 20 ]. In the current report, the patient received liposomal Amphotericin B for one month in combination with surgical debridement, resulting in the survival of the patient. Afterward, she managed to take posaconazole for four weeks. 

However, in the study performed by Gulati *et al*. [ 14 ], the patient died after treatment with deoxycholate and amphotericin B.

In AFST analysis, *S. racemosum* showed good susceptibility to Amphotericin B, 0.032 µg/ml; luliconazole, 0.25 µg/ml; posaconazole, 0.5 µg/ml; and itraconazole, 0.5 µg/ml, respectively. The AFST evaluation for *S. racemosum* reported by Raju *et al*. (2020) also demonstrated a low MIC value for posaconazole (0.06 µg/ml), Amphotericin B, and itraconazole (0.5 µg/ml) [ 21 ]. Chowdhary *et al*. [ 22 ] previously showed that *S. racemosum* isolates from rhino-cerebral and cutaneous samples had MICs of 0.047 μg/ml for Amphotericin B, and 16 μg/ml for itraconazole and voriconazole [ 22 ]. 

In another study by Singh *et al*. [ 23 ] four clinical isolates of *S. racemosum* originated from subcutaneous and invasive infection showed low MIC values for amphotericin B (AMB) (GM MIC, 0.218 μg/ml), itraconazole (ITR) (GM MIC, 0.178 μg/ml), posaconazole (POS) (GM MIC, 0.325 μg/ml), and terbinafine (TER) (GM MIC, 0.071 μg/ml).

Vitale *et al*. [ 24 ] calculated the MIC for one of the *S. racemosum* isolates obtained from skin samples, including AMB (0.25 μg/ml), ITR (0.03 μg/ml), POS (0.06 μg/ml), and TER (> 4μg/ml) [ 24 ]. 

Our results indicated that luliconazole (with MIC=0.25 µg/ml) had better activity against *S. racemosum* compared with posaconazole (MIC= 0.5 µg/ml), isavuconazole (MIC= 1 µg/ml), and voriconazole (MIC >16 µg/ml).

Omran *et al*. [ 25 ] and Abastabar *et al*. [ 26 ] recently noted that luliconazole had superiority against *Aspergillus flavus*, *A. fumigatus*, and *Fusarium* strains in comparison with the common antifungal drugs.

## Conclusion

Our study emphasized the pathogenic role of *S. racemosum* as a possible agent of mucormycosis in diabetic patients with COVID-19. To the best of our knowledge, this is the first description of *S. racemosum* infection in COVID-19 patients worldwide. Early detection of *S. racemosum* using precise methods such as PCR sequencing is uncommon, but treatment with antifungal drugs such as amphotericin B or posaconazole as well as surgical debridement is the cornerstone of proper management of CAM due to *S. racemosum*.

## Acknowledgments

The written informed consent was obtained from the patient. This research was financially supported (grant number: 9707034) by the Babol University of Medical Science and Mazandaran University of Medical Sciences (grant number: 5747).

## Authors’ contribution

M.T.A., M.A., and S.M. were responsible for manuscript writing and editing. M.B., M.N., L.F., S.Y., B.N., and A.T. were involved in the management of patients during the hospital stay. M.T.H., J.J., and I.H. thoroughly reviewed the manuscript. All authors have made a substantial contribution to the current case report and approved the final manuscript.

## Conflicts of interest

The authors reported no conflicts of interest.

## Financial disclosure

No financial interests related to the material of this manuscript have been declared.

## Ethical Considerations

This study was approved by the ethics committee of the Babol University of Medical Science (IR.MUBABOL.HRI.REC.1400.117) and Mazandaran University of Medical Science (IR.MAZUMS.REC. 1398.1136), respectively.
